# Tree‐temporal scan statistics for safety signal detection in vaccine clinical trials

**DOI:** 10.1002/pst.2391

**Published:** 2024-04-15

**Authors:** François Haguinet, Fabian Tibaldi, Christophe Dessart, Andrew Bate

**Affiliations:** ^1^ Global Safety GSK Wavre Belgium; ^2^ Global Statistics GSK Rixensart Belgium; ^3^ Global Safety GSK Middlesex UK; ^4^ Department of Non‐Communicable Disease Epidemiology London School of Hygiene and Tropical Medicine London UK; ^5^ Department of Medicine NYU Grossman School of Medicine New York New York USA

**Keywords:** safety, statistics, tree‐temporal scan, vaccines

## Abstract

The evaluation of safety is critical in all clinical trials. However, the quantitative analysis of safety data in clinical trials poses statistical difficulties because of multiple potentially overlapping endpoints. Tree‐temporal scan statistic approaches address this issue and have been widely employed in other data sources, but not to date in clinical trials. We evaluated the performance of three complementary scan statistical methods for routine quantitative safety signal detection: the self‐controlled tree‐temporal scan (SCTTS), a tree‐temporal scan based on group comparison (BGTTS), and a log‐rank based tree‐temporal scan (LgRTTS). Each method was evaluated using data from two phase III clinical trials, and simulated data (simulation study). In the case study, the reference set was adverse events (AEs) in the Reference Safety Information of the evaluated vaccine. The SCTTS method had higher sensitivity than other methods, and after dose 1 detected 80 true positives (TP) with a positive predictive value (PPV) of 60%. The LgRTTS detected 49 TPs with 69% PPV. The BGTTS had 90% of PPV with 38 TPs. In the simulation study, with simulated reference sets of AEs, the SCTTS method had good sensitivity to detect transient effects. The LgRTTS method showed the best performance for the detection of persistent effects, with high sensitivity and expected probability of type I error. These three methods provide complementary approaches to safety signal detection in clinical trials or across clinical development programmes. All three methods formally adjust for multiple testing of large numbers of overlapping endpoints without being excessively conservative.

## 1 INTRODUCTION

The evaluation of the safety of interventions is critical in all clinical trials. In phase I and phase II of clinical development, when trials are typically exploratory with small sample sizes, the evaluation of safety is mainly sensitive to the expression of significant toxicity in individual patients that was not anticipated in pre‐clinical studies. In later phase trials, larger sample sizes allow wider exploration of the safety profile of investigational products.^1^ Periodic analysis of the accumulated safety information during the clinical development programme (CDP) of a new drug or vaccine is paramount for the evaluation of the risk of harm to study participants.^2^, ^3^, ^4^

Periodic analyses have proven useful for holistic reviews of the most up‐to‐date safety data collected throughout a CDP. However, the conclusions based on periodic reports data are not without limitations: they mainly focus on serious adverse events (SAEs), and these are reported as Medical Dictionary for Regulatory Activities (MedDRA) preferred terms (PTs)^5^; furthermore, administration of non‐study products or protocol violations for exposed participants are not quantitatively accounted for in a systematic way in these analyses, even though these data are frequently collected during clinical trials.

Pooling of safety data or secondary data analysis of safety outcomes with meta‐analysis from multiple, late phase randomised clinical trials (RCTs) (or at the level of an entire CDP) are sometimes conducted to better describe the safety profile of an investigational product.^6^ During studies, various sequential quantitative analysis approaches have been explored for hypothesis generation and safety surveillance in RCTs^7^ and also evaluated in other data sources.^8^ Data mining techniques have also been used as surveillance approaches to generate safety hypotheses,^9^, ^10^ although there is no preferred method for data mining, and the performance testing of different approaches has been limited.

We aimed to evaluate the performance of three complementary data mining methods, separately and together for routine quantitative signal detection in safety data obtained during the clinical development of vaccines: the self‐controlled tree‐temporal scan (SCTTS), a tree‐temporal scan based on group comparison (BGTTS), and a log‐rank based tree‐temporal scan (LgRTTS). The LgRTTS is a novel statistical method, developed in the context of this work to address long‐term analyses perturbed by withdrawals of subjects before the end of the analysis period. We evaluated each method using both existing data from completed clinical trials and simulated data.

## 2 DESCRIPTION OF THE PROBLEM

The quantitative analysis of safety data in clinical trials poses well‐established statistical challenges,^1^ which can be considerable at the level of an entire CDP (pooled data).^6^ An estimand describes the true population quantity of interest that needs to be estimated to answer a precise clinical question on a treatment effect^11^, ^12^ and is conceptually very useful when the primary research question (or even secondary) can be precise, for example in traditional well‐constructed late phase efficacy trials. Unkel et al.^13^ extended this conceptual framework with safety estimands which focus on quantities to estimate to summarise AE data in clinical trials. While in theory increasing precision in clarity around a safety related question of interest is attractive, in practice the definition of estimands for safety objectives is challenging because the related research question is inherently exploratory, encompassing many safety outcomes and treatment effects with much uncertainty across the five attributes of estimands.^12^ Even when the treatment and the targeted population of safety estimands are usually well defined in the study protocol, the other attributes often cannot be precisely defined.

### 2.1 Safety endpoints

Safety data are collected and analysed in predefined periods, for instance 7 days after vaccination for solicited AEs, 30 days for unsolicited AEs or 180 days for SAEs. However, the effect of exposure to the investigational product can be persistent or transient, and may not match with these predefined periods.^14^ The increased risk of febrile convulsions 5–12 days after measles‐mumps‐rubella‐varicella immunization^15^ and the higher risk of intussusception within 7 days of vaccination with rotavirus vaccine^16^ are well‐known examples of inconsistencies between surveillance periods and periods with higher risk due to exposure. If the period is too short or overly long, an association may be undetected. If the exposure has a transient effect on the risk of an AE but the impacted period is unknown, several plausible risk periods need to be tested.

Another challenge is that AEs are usually classified as MedDRA PTs. There can be a risk of decrease in power if events for the same medical context (i.e., signs, symptoms, diagnoses, syndromes, physical findings, laboratory, and other physiologic test data associated with the condition of interest) are not optimally grouped together under the condition of interest using customised or standardised MedDRA queries (SMQs) or according to the MedDRA hierarchy.

The range of possible adverse events (AEs), how they manifest themselves, their timing after exposure and their frequency is variable and difficult to fully anticipate. Designing clinical trials to evaluate all safety endpoints with sufficient power to be conclusive is impossible. If some specific safety endpoints identified a priori are a focus for evaluation with well‐defined estimands, the evaluation of other safety endpoints in the same study becomes more of a data mining issue,^9^ with the aim of generating statistical alerts for hypothetical safety issues. The intent of estimands is to add more clarity/specificity to endpoint definitions, which fits with hypothesis testing, whereas this level of specificity is not appropriate for data mining where the aim is to identify hypothesis for further investigation. Therefore, some flexibility is needed in the definition of the endpoints for safety related hypothesis generation.

### 2.2 Strategies for intercurrent events

In the context of this work, a principal stratum strategy consisted of considering only participants not withdrawn before the planned analysis period. The while‐on‐treatment strategy consisted of considering events and time‐at‐risk only until withdrawal during the analysis period. The most usual strategy for safety data in clinical trials is the while‐at‐risk,^17^ which is a modified treatment policy strategy in which the intention‐to‐treat approach is not respected because the treatment group of each individual corresponds to the treatment actually administered, and the data are analysed according to the schedules planned in the protocol, no matter the occurrence of intercurrent events. The period at‐risk refers to the period assumed to be influenced by the exposure as predefined in the protocol, for instance 30 days post‐vaccination for the unsolicited AEs. It is hard to define precisely and appropriately the right strategy for each safety outcome in a hypothesis generation framework. And yet, the different strategies may have various impact on the estimated population‐level summary depending on the assumptions behind it, justifying the need to test a range of alternatives.

### 2.3 Population‐level summary

The estimand attributes should be considered interrelated.^18^ The flexibility needed in the safety endpoint definitions and in the strategies for intercurrent events in a hypothesis generation/surveillance framework requires flexible methods to generate population‐level summaries.

Most safety evaluations in clinical trials consist of a descriptive analysis of all AEs, with computation of incidence proportions or exposure‐adjusted incidence rates of subjects experiencing each event within a predefined exposure period within exposed and comparator groups, according to the while‐at‐risk or while‐on‐treatment strategies for intercurrent events, respectively. Confidence intervals of incidences are sometimes computed to reflect the uncertainty of the estimate. When p‐values are obtained from exploratory statistical comparisons, they are usually used to rank AEs worthy of further attention.^1^

While the analysis of numerous safety endpoints leads to difficulties in controlling the probability of false positives resulting from the multiplicity of the tests,^19^ there is no consensus on whether adjustment for multiplicity should be performed.^1^, ^4^, ^20^ The issue of multiplicity might be attempted to be controlled for by adapting the significance levels via approaches such as the Bonferroni correction or the less conservative control of the false discovery rate (FDR).^21^, ^22^ However, both methods assume independence across outcomes and are too conservative in case of correlated outcomes.^23^, ^24^

In the analysis of pooled data from several studies within the CDP of an investigational drug or vaccine, the differences inherent to the populations studied (i.e., age‐groups, health status, ethnicities, etc.), randomisation schemes or group distributions (i.e., 1:1 vs. 1:2), schedules, formulations, and co‐administered products pose additional challenges.^1^

Scan statistical methods have been tested in post‐marketing safety data for signal detection^25^, ^26^, ^27^ but to our knowledge, have not been applied to clinical data. Since tree‐based and temporal scan statistical methods allow for some flexibility in the endpoint definitions and control the probability of false positives (i.e., type I error) by adjustment for multiple testing with Monte‐Carlo simulations, we considered their possible use for routine periodic assessment of safety information collected in a product CDP and at a study level, particularly for large phase III studies. Temporal scan methods do not require pre‐defined exposure periods for all outcomes. The self‐controlled temporal scan also potentially addresses the difficulties of maintaining the randomisation for pooled data, or the monitoring of blinded data during ongoing trials. Therefore, we aimed to evaluate scan statistical methods for routine quantitative signal detection using clinical trial data of vaccines at the study and CDP levels.

## 3 VARIABLE APPROACHES TO MULTIPLICITY OF SAFETY ENDPOINTS

Several regulatory guidances address the analysis of safety data in clinical trials and illustrate the challenges and lack of consensus in the analysis of safety data.^1^, ^20^, ^28^ The ICH E9 Statistical principles for clinical trials^1^ notes that when statistical tests are used, adjustment for multiplicity to manage the type I error is useful, even if the type II error is of more concern. “Points to Consider on Multiplicity Issues in Clinical Trials” published by the European Medicines Agency Committee for Proprietary Medicinal Products mentions the use of statistical tests as a flagging device and considers multiplicity adjustment as counterproductive.^20^ “Management of Safety Information from Clinical Trials” published by the Council for International Organisations of Medical Sciences VI working group recognises the difficulty of analysis safety data in clinical trials, mentioning some methods but making no clear recommendations.^4^

A recent review highlights the wide variety of approaches used to analyse multiple safety outcomes, depending whether they are emerging safety concerns or pre‐defined outcomes.^29^ These include visual summary methods such as volcano plots, heat maps, and forest plots; hypothesis testing methods including FDR p‐value adjustments,^21^, ^22^, ^30^ a variety of methods that aim to combine multiple events for safety profile evaluation (for instance, tests using exact permutation distribution), and methods providing decision making probabilities such as Bayesian hierarchical models.^31^, ^32^

## 4 SCAN STATISTICAL METHODS

### 4.1 Self‐controlled tree‐temporal scan

The SCTTS detects transient associations between exposure to a product and a wide range of safety related endpoints in exposed persons.^27^ The analysis period for each exposed person is divided into exposure and control periods, each person serving as their own control (i.e., *self‐controlled*). Consequently, fixed covariates (i.e., study, gender) are controlled for confounding.

The analysis period, including control period(s), is preferably entirely positioned after the exposure to avoid biases due to conditions modifying the probability of exposure or its timing, as it was documented for the self‐controlled case series analysis.^33^ To the extent possible, the analysis period should be long enough to include control periods less likely to be influenced by the exposure, which also increases the power of the analysis. On the other hand, long analysis periods are more subject to time‐varying confounders and a good trade‐off should be found.

The *tree scan* consists of a sequential investigation of each outcome at each hierarchical level of a dictionary of diagnoses, called nodes. *Temporal scan* means that the length and position of the exposure period varies within the analysis period according to pre‐specified rules, such as the range of the length and increments in shift, and extension of the exposure period.

Related to the estimand framework,^13^ the intercurrent events occurring during the analysis period can be ignored, adopting the while‐at‐risk strategy described in Fu et al.^17^ The principal stratum strategy^12^ can also be adopted, the principal stratum including subjects followed for the entire analysis period.

For each step or couple (node, exposure period) of the tree‐temporal scan, the log likelihood ratio (LLR) test statistic is computed as:

$$\;\mathrm{LLR}=\ln\frac{{\left(\frac{a}{a+b}\right)}^{a}{\left(\frac{b}{a+b}\right)}^{b}}{{\left(\frac{r}{r+c}\right)}^{a}{\left(\frac{c}{r+c}\right)}^{b}}\;I\left(\left(\frac{a}{a+b}\right)>\left(\frac{r}{r+c.}\right)\right),$$

where *a* is the number of node cases with time‐to‐onset (TTO) in the exposure period, *b* is the number of node cases with TTO in the control period, *r* is the length of the exposed period, and *c* is the length of the control period.

The *I*() is the indication function ensuring that we are looking for an excess risk in the exposure period.

For each node, the most likely exposure period is the one with the highest LLR; however, this does not mean there is an excess risk during this period. The entire tree‐temporal scan process is summarised in the test statistic, which is obtained by maximising LLR over all nodes and exposure periods. Its statistical distribution needs to be estimated from Monte‐Carlo simulations,^34^ assuming a uniform distribution of the TTO under the null hypothesis (no association, H0). The TTOs of all events are simulated from a uniform distribution within the analysis period many times. The highest LLR over the entire tree‐temporal scan process in each simulation is included in the empirical distribution. For each node, a p‐value can be computed from the estimated distribution, which is adjusted for multiplicity of testing across all nodes and exposure windows.

Because the TTO distribution under H0 can be perturbed by some factors, for instance a weekly periodicity or a period of enhanced monitoring, a conditional version of the statistic can be used. However, in this case, the expected TTO distribution under H0 is estimated from all AE cases reported and may be hampered by some frequent AEs clustered during a specific interval, for example, local reactogenicity, which typically occurs in the first 24 h after vaccination. The Poisson generalised LLR is:

$${\mathrm{LLR}}_{\text{Cond}}=\ln\frac{{\left(\frac{a}{a+b}\right)}^{a}{\left(\frac{\left(R+C\right)-a}{R+C}\right)}^{\left(\left(R+C\right)-a\right)}}{{\left(\frac{R}{R+C}\right)}^{a}{\left(\frac{\left(R+C\right)-\left(\left(a+b\right)\left(\frac{R}{R+C}\right)\right)}{R+C}\right)}^{\left(\left(R+C\right)-a\right)}}\;I\left(\left(\frac{a}{a+b}\right)>\left(\frac{R}{R+C}\right)\right),$$

with *a* being the number of node cases with TTO in the exposed period, *b* being the number of node cases with TTO in the control period, *R* being the number of cases in exposure period among all cases (over the whole tree), and *C* being the number of cases in the control period among all cases.

The empirical distribution is computed using Monte‐Carlo simulations.

### 4.2 Between group tree‐temporal scan

The BGTTS is based on the tree scan method,^25^ and is similar to SCTTS but is not self‐controlled, the comparator being another study group. The BGTTS can detect transient or persistent differences between two groups. The intercurrent events can be addressed with the same strategies as SCTTS. We added the temporal scan to the tree scan, otherwise the same exposure period would be considered for all safety endpoints. This includes those with a potentially transient effect of exposure, or those with a specific surveillance period not corresponding to the analysis period. As before, the test statistic is obtained by maximising LLR over the entire tree‐temporal scan process:

$$\;\mathrm{LLR}=\ln\frac{{\left(\frac{a}{a+b}\right)}^{a}{\left(\frac{b}{a+b}\right)}^{b}}{{\left(\frac{E}{E+C}\right)}^{a}{\left(\frac{C}{E+C}\right)}^{b}}\;I\left(\left(\frac{a}{a+b}\right)>\left(\frac{E}{E+C}\right)\right),$$

where *a* is the number of node cases in the exposed group, *b* is the number of node cases in the control group, *E* is the number of exposed subjects, and *C* is the number of subjects in the control group.

The LLR can be adjusted for fixed covariates such as study or sex by stratification. The expected group distribution of the cases within each stratum corresponds to the group distribution of its subjects. Multiplicity is addressed using Monte‐Carlo simulations of the groups according to the probability of exposure defined by the design of the study.

### 4.3 Log‐rank based tree‐temporal scan

The use of time‐to‐event methodology for the analyses of AEs has been recommended since a long time.^10^, ^13^, ^35^, ^36^ According to the ICH‐E9 and as cited by Wang^36^ “The popular practice in reporting AE to regulatory agencies is using AE proportion and incidence rate. When the drug is a long‐term treatment and a substantial proportion of intercurrent events such as treatment withdrawal or death is expected, “survival analysis methods should be considered, and cumulative adverse event rates calculated in order to avoid the risk of underestimation”.” We developed this new scan statistical method in the context of this work. It allows performance of a tree‐temporal scan for the comparison of the TTO distribution between the exposed and control groups without assumptions about the baseline TTO distribution. The method supports the inclusion of subjects withdrawn before the end of the analysis period (prematurely censored data) and therefore the analysis of long‐term exposure effects. This approach corresponds to the while‐on‐treatment strategy for intercurrent events in the estimands framework.^12^, ^13^

It is similar to BGTTS, but instead of a LLR test based on a binomial distribution, we used the log‐rank test for the comparison of the distribution of TTO between groups.^37^ The log‐rank test statistic follows a chi‐squared distribution with one degree of freedom regardless of the number of events observed or the length of follow‐up. As for the BGTTS method, this makes the estimation of an empirical distribution of the maximised log‐rank statistics realistic across the tree nodes and the exposure periods under H0 (no exposure effect) using Monte‐Carlo simulations, addressing the multiplicity similarly to BGTTS.

We performed simulations at an aggregate level within the life‐tables, randomly re‐attributing the events between the groups at each timepoint according to a binomial distribution. The probability of exposure is the proportion of exposed subjects among subjects remaining in the study (not previously censored and without a previous event), and the number of trials is the number of events at the considered timepoint. This approach was preferred to permutations between groups to gain computational efficiency.

The temporal scan was included because the log‐rank test assumes proportional hazards during the analysis period.^18^, ^37^ For transient effects, this proportionality is sometimes met only during part of a subject's follow‐up. For instance, with live‐attenuated vaccines, some AEs only appear during viral replication, which occurs around a week after vaccination. A stratified version of the log‐rank statistic can be used to address confounding factors.

## 5 ILLUSTRATIVE CASE STUDY

The tree‐temporal scan methods were tested on the pooled final data from the ZOE‐50 and ZOE‐70 trials—two similarly designed, pivotal phase III efficacy studies that evaluated the adjuvanted recombinant zoster vaccine (RZV, *Shingrix*, GSK) administered as two doses 2 months apart.^38^, ^39^ As a reference, asymptotic tests for stratified person‐time data^24^ (SPT test) with FDR (SPT‐FDR) and Bonferroni (SPT‐Bon) multiplicity adjustments were also tested.

The aims of this illustrative case study were (1) to evaluate a comprehensive analysis of the safety data collected in pre‐existing clinical trials data based on the scan statistical methods to allow for data‐driven outcomes definitions (tree scan) and data‐driven exposure periods (temporal scan), and (2) to compare the methods when applied to similar estimands within the constraints of the retrospective use of already completed clinical trials, to provide information on their redundancy or complementarity (see Table 1 for estimand definitions).

**TABLE 1:** Safety estimands in the illustrative use case (presented similarly to Wang et al.)^36^

**Question of interest**: Are there statistical alerts for any transient increased risk associated with the experimental vaccine within 20 days (Day 0‐Day 19) after each vaccination with respect to the incidence of solicited AEs, unsolicited AEs, SAEs and pIMDs observed within 30 days (Day 0‐Day 29) after each vaccination?	
Treatment	The experimental vaccine
Variable	Occurrence of solicited AEs, unsolicited AEs, SAEs and pIMDs observed within 30 days (Day 0‐Day 29) after each vaccination
Intercurrent events	Premature withdrawals were intercurrent events of interest and were handled with a while‐at‐risk strategy. Specifically, all exposed participants were considered. Withdrawals within the analysis period were ignored
Population Level Summary	P‐values <0.1 for the tests of any period of higher risk within 20 days after each dose of the experimental vaccine using SCTTS
**Question of interest**: Are there statistical alerts for any transient or persistent increased risk associated with the experimental vaccine versus the placebo with respect to the incidence or time‐to‐event distributions of solicited AEs, unsolicited AEs, SAEs and pIMDs observed within 30 days (Day 0‐Day 29) after vaccinations?	
Treatment	The experimental vaccine and placebo control
Variable	Occurrence of solicited AEs, unsolicited AEs, SAEs and pIMDs observed within 30 days (Day 0‐Day 29) after each vaccination
Intercurrent events	Premature withdrawals were intercurrent events of interest and were handled in three ways depending on analyses: While‐at‐risk strategy for the BGTTS after each dose. Specifically, all exposed participants were considered. Withdrawals within the analysis period were ignored Principal stratum strategy for the BGTTS in combined analysis periods of both doses: only participants who received two doses and not withdrawn before day 30 after the second dose were considered While on treatment strategy for the SPT‐FDR, SPT‐Bon and LgRTTS. Specifically, withdrawals within the analysis period truncated the time at risk of individuals
Population Level Summary	P‐values <0.1 for the tests of a higher risk in the experimental vaccine than the placebo using BGTTS, SPT‐FDR, SPT‐Bon and LgRTTS within 30 days after each dose separately and BGTTS within the combined 30 days periods after both doses
**Question of interest**: Are there statistical alerts for any transient or persistent increased risk associated with the experimental vaccine versus the placebo with respect to the incidence or time‐to‐event distributions of solicited AEs, unsolicited AEs, SAEs and pIMDs observed within 420 days (Day 0‐Day 419) after vaccination?	
Treatment	The experimental vaccine and placebo control
Variable	Occurrence of solicited AEs, unsolicited AEs observed within 30 days (Day 0‐Day 29) after each vaccination, and SAEs and pIMDs observed within 420 days (Day 0‐Day 419) after first dose
Intercurrent events	Premature withdrawals were intercurrent events of interest and were handled with a while on treatment strategy. Specifically, withdrawals within the analysis period truncated the time at risk of individuals
Population Level Summary	P‐values <0.1 for the tests of a higher risk in the experimental vaccine than the placebo using SPT‐FDR, SPT‐Bon and LgRTTS within 420 days after first dose

*Note*: For all estimands the *targeted population* was the same, corresponding to the inclusion/exclusion criteria in the protocol.

Abbreviations: AE, adverse event; BGTTS, between group tree‐temporal scan; ICE, intercurrent events; LgRTTS, log‐rank based tree‐temporal scan; pIMD, potential immune mediated disease; SAE, serious adverse event; SCTTS, self‐controlled tree‐temporal scan; SPT‐Bon, asymptotic test for stratified person‐time data with Bonferroni correction; SPT‐FDR, asymptotic test for stratified person‐time data with false discovery rate correction.

### 5.1 Data

The ZOE‐50 and ZOE‐70 studies were identically designed and only differed in the age range at the time of the first vaccination: ZOE‐50 included subjects aged ≥50 years, while ZOE‐70 included subjects aged ≥70 years. Both were randomised, observer‐blind, placebo‐controlled, multi‐centre studies. Each study had two groups randomised 1:1 to receive two doses of either the RZV vaccines or placebo. A subset of subjects was randomised to a reactogenicity subset and recorded solicited AEs on diary cards from Day 0 to Day 6 after each vaccination. 30‐day diary cards were completed by all subjects to record unsolicited AEs from Day 0 to Day 29 after each vaccination. SAEs were collected from Month 0 to Month 14 and fatal SAEs, related SAEs, and potential immune‐mediated diseases (pIMDs)^40^ were captured during the entire study period in all subjects.

### 5.2 Analyses

Three questions of interest were addressed in the analyses from the narrowest to broadest in term of safety data and exposure effect covered. Are there statistical alerts for increased risk associated with the experimental vaccine with respect to safety events (solicited AEs, unsolicited AEs, SAEs and pIMDs)?

1. Any transient increased risk within 20 days (Day 0–Day 19) with respect to the incidence of safety events observed within 30 days (Day 0–Day 29) after vaccination.
2. Any transient or persistent increased risk with respect to the time‐to‐event distributions or incidence of safety events observed within 30 days (Day 0–Day 29) after vaccination.
3. Any transient or persistent increased risk with respect to the time‐to‐event distributions or incidence of safety events observed within 420 days (Day 0–Day 420) after vaccination.

The estimand attributes corresponding to each of these questions are described in Table 1. All the estimands targeted the study populations according to the inclusion/exclusion criteria of the protocol. The SCTTS within the 30‐day periods after each RZV dose separately was used to answer the first question. The second question was addressed by using: (1) the BGTTS, LgRTTS, SPT‐FDR, and SPT‐Bon for the 30‐day period after each RZV dose separately, (2) the BGTTS for the combined 30‐day periods after both RZV doses (referred to as *combined doses* hereafter). The third question was addressed with the LgRTTS, SPT‐FDR, and SPT‐Bon for the 420‐day period after RZV dose 1.

The hierarchical tree for the tree scan was based on MedDRA coding and used three levels: PT, high‐level term (HLT), and system organ class (SOC). MedDRA queries corresponding to pIMDs were also added to the tree.

To our knowledge the SCTTS has been mainly used in observational studies,^27^, ^41^ allowing for more distant control periods and less influenced by the exposure. In the clinical trials considered for our testing, the analysis periods were limited by the periods of collection of unsolicited AEs. The temporal scan of the SCTTS was performed by day of increment and moved within the first 20 days after vaccination. The next 10 days (Days 20–29) were a fixed control period.

In the analyses within 30 days of enhanced monitoring of unsolicited AEs after vaccination, the temporal scan for the BGTTS and the LgRTTS was performed by day of increment and moved within the 30‐day period. In the combined doses analysis, subjects had an event if they experienced an event either after one of the doses or after both. In the long‐term analyses, the temporal scan in the LgRTTS was performed by week of increment and moved within the 420‐day analysis period, and exposure periods starting later than 84 days post dose 1 were not considered.

The performance of each method was evaluated by calculating the sensitivity (Se) and the positive predictive value (PPV) at a significance level of 10%.^42^ The reference set of positive controls was the list of 72 AEs (given as MedDRA PTs or synonyms) in the Reference Safety Information of RZV.^43^, ^44^ The Se was only computed at the level of the PT because its computation requires a set of positive controls defined a priori. The statistical alerts generated at all tree levels were reviewed by safety experts and classified into three categories: *True positive* — when the statistical alert clearly corresponds to an identified AE; *Potentially true —* when the statistical alert could make sense for biological or other reasons; and *False positive* — when the statistical alert was clearly false due to biological implausibility. On this basis, the PPV was estimated once considering *Potentially true* statistical alerts as true positives, and once considering them as false positives.

All analyses were performed using customised programmes in the SAS software V.9.4.^45^ An example of the code for the SCTTS is included in the supplementary material.

### 5.3 Results

Across both studies, 15,455 participants received RZV and 15,468 received placebo.

#### 5.3.1 Detection of transient effects within the 20‐day periods after vaccination

Among the 72 listed PTs, 20 were detected by SCTTS post dose 1 and post dose 2 (Tables 2 and 3). One additional listed PT was detected post dose 1 and same post dose 2 resulting in 22 listed PTs detected with a transient increased incidence within 20 days after any dose.

**TABLE 2:** Sensitivity based on listed MedDRA PTs and PPVs for all analyses (alpha = 0.1) (illustrative case study).

Exposed period^a^	Method	Sensitivity^b^	PPV	
			True only^c^	With potential^d^
20 days post‐dose 1	SCTTS	29.17% (21/72)	60.15% (80/133)	67.67% (90/133)
20 days post‐dose 2	SCTTS	29.17% (21/72)	55.80% (77/138)	63.04% (87/138)
30 days post‐dose 1	SPT‐Bon	19.44% (14/72)	89.74% (35/39)	92.31% (36/39)
	SPT‐FDR	20.83% (15/72)	86.00% (43/50)	88.00% (44/50)
	BGTTS	19.44% (14/72)	90.48% (38/42)	92.86% (39/42)
	LgRTTS	19.44% (14/72)	88.64% (39/44)	90.91% (40/44)
30 days post‐dose 2	SPT‐Bon	20.83% (15/72)	83.02% (44/53)	86.79% (46/53)
	SPT‐FDR	22.22% (16/72)	62.34% (48/77)	76.62% (59/77)
	BGTTS	19.44% (14/72)	75.86% (44/58)	77.59% (45/58)
	LgRTTS	22.22% (16/72)	69.01% (49/71)	76.06% (54/71)
30 days post‐dose 1 or 2^e^	BGTTS	22.22% (16/72)	70.15% (47/67)	76.12% (51/67)
420 days post‐dose 1	SPT‐Bon	20.83% (15/72)	82.35% (42/51)	88.24% (45/51)
	SPT‐FDR	22.22% (16/72)	68.49% (50/73)	79.45% (58/73)
	LgRTTS	20.83% (15/72)	78.95% (45/57)	84.21% (48/57)
All data	SPT‐Bon	20.83% (15/72)	78.95% (45/57)	84.21% (48/57)
	SPT‐FDR	22.22% (16/72)	61.18% (52/85)	75.29% (64/85)
	Any Scan Statistics	30.56% (22/72)	54.19% (84/155)	60.65% (94/155)

Abbreviations: BGTTS, tree‐temporal scan based on group comparison; LgRTTS, log‐rank based tree‐temporal scan; MedDRA, Medical Dictionary for Regulatory Activities; PPV, positive predictive value; PT, preferred term; SCTTS, self‐controlled tree‐temporal scan; SPT‐Bon, asymptotic test for stratified person‐time data with Bonferroni correction; SPT‐FDR, asymptotic test for stratified person‐time data with false discovery rate correction.

^a^ Temporal scan period for BGTTS, LgRTTS and SCTTS.

^b^ The sensitivity is relative to the 72 MedDRA PT corresponding to the adverse events in the Reference Safety Information of RZV.

^c^ Computation of PPV including only confirmed true positives.

^d^ Computation of PPV including confirmed and potential true positives.

^e^ Pooled data from 30 days post‐dose 1 and 30 days post‐dose 2 in persons exposed to both doses.

**TABLE 3:** Agreement of true positives between analyses (illustrative case study). Statistical alerts in the list of MedDRA PTs or synonyms in the Reference Safety Information of RZV. The colour scale from yellow to green corresponds to the increasing number of true positives.

	BGTTS D1	LgRTTS D1	SPT‐FDR D1	SPT‐Bon D1	SCTTS D1	All TTS D1	BGTTS D2	LgRTTS D2	SPT‐FDR D2	SPT‐Bon D2	SCTTS D2	All TTS D2	BGTTS D1&2	LgRTTS LFU	SPT‐FDR LFU	SPT‐Bon LFU	All TTS	All SPT‐FDR	All SPT‐Bon
BGTTS D1	14																		
LgRTTS D1	14	14																	
SPT‐FDR D1	14	14	15																
SPT‐Bon D1	14	14	14	14															
SCTTS D1	14	14	15	14	21														
All TTS D1	14	14	15	14	21	21													
BGTTS D2	13	13	14	13	14	14	14												
LgRTTS D2	13	13	14	13	16	16	14	16											
SPT‐FDR D2	14	14	15	14	16	16	14	15	16										
SPT‐Bon D2	14	14	15	14	15	15	14	14	15	15									
SCTTS D2	14	14	15	14	20	20	14	16	16	15	21								
All TTS D2	14	14	15	14	20	20	14	16	16	15	21	21							
BGTTS D1&2	14	14	15	14	16	16	14	15	16	15	16	16	16						
LgRTTS LFU	14	14	15	14	15	15	14	14	15	15	15	15	15	15					
SPT‐FDR LFU	14	14	15	14	16	16	14	15	16	15	16	16	16	15	16				
SPT‐Bon LFU	14	14	15	14	15	15	14	14	15	15	15	15	15	15	15	15			
All TTS	14	14	15	14	21	21	14	16	16	15	21	21	16	15	16	15	22		
All SPT‐FDR	14	14	15	14	16	16	14	15	16	15	16	16	16	15	16	15	16	16	
All SPT‐Bon	14	14	15	14	15	15	14	14	15	15	15	15	15	15	15	15	15	15	15

Based on expert review, and therefore potentially including statistical alerts at all tree levels, 80 and 77 true positives were detected after dose 1 and dose 2, respectively, with 73 in common (Table 4). For both doses, eight statistical alerts at HLT level and one at SOC level had no alerts in their descendants in the hierarchical tree.

**TABLE 4:** Agreement of true positives between analyses (illustrative case study). Statistical alerts classified as true positives by expert review. The colour scale from yellow to green corresponds to the increasing number of true positives.

	BGTTS D1	LgRTTS D1	SPT‐FDR D1	SPT‐Bon D1	SCTTS D1	All TTS D1	BGTTS D2	LgRTTS D2	SPT‐FDR D2	SPT‐Bon D2	SCTTS D2	All TTS D2	BGTTS D1&2	LgRTTS LFU	SPT‐FDR LFU	SPT‐Bon LFU	All TTS	All SPT‐FDR	All SPT‐Bon
BGTTS D1	38																		
LgRTTS D1	38	39																	
SPT‐FDR D1	37	37	43																
SPT‐Bon D1	35	35	35	35															
SCTTS D1	38	39	43	35	80														
All TTS D1	38	39	43	35	80	80													
BGTTS D2	35	36	37	32	44	44	44												
LgRTTS D2	37	38	39	34	49	49	44	49											
SPT‐FDR D2	37	38	40	35	48	48	43	47	48										
SPT‐Bon D2	37	38	40	35	44	44	41	43	44	44									
SCTTS D2	38	39	43	35	73	73	44	49	48	44	77								
All TTS D2	38	39	43	35	73	73	44	49	48	44	77	77							
BGTTS D1&2	38	39	40	35	47	47	43	46	46	44	47	47	47						
LgRTTS LFU	38	39	40	35	45	45	41	44	44	43	45	45	45	45					
SPT‐FDR LFU	38	39	42	35	50	50	43	47	47	44	50	50	47	45	50				
SPT‐Bon LFU	37	37	40	35	42	42	38	41	42	41	42	42	42	42	42	42			
All TTS	38	39	43	35	80	80	44	49	48	44	77	77	47	45	50	42	84		
All SPT‐FDR	38	39	43	35	52	52	44	48	48	44	52	52	47	45	50	42	52	52	
All SPT‐Bon	37	38	40	35	45	45	41	44	45	44	45	45	45	44	45	42	45	45	45

#### 5.3.2 Detection of transient and persistent effects within the 30‐day periods after vaccination

Based on expert review and considering only statistical alerts at SOC and HLT levels without alerts in their descendants, two statistical alerts at the SOC level and two statistical alerts at the HLT level were detected by the BGTTS after dose 2 and in combined doses. The same alerts at SOC level and one of the alerts at HLT level were detected by the LgRTTS method after dose 2.

The LgRTTS seemed a bit more sensitive than the BGTTS, with two additional listed PTs detected after dose 2 (Tables 2 and 3) and, when including statistical alerts at all tree levels based on expert review, one additional true positive after dose 1 and five after dose 2 (Table 4). Similar observations were made when including potentially true positives (Table 5). All alerts from the BGTTS after each dose were also detected by LgRTTS, with two additional post dose 1 and 13 additional post dose 2 (Table 6). This finding may be due to the higher number of persons withdrawn from the study during the 30‐days period after dose 2 in both exposure groups, which is better addressed with a time‐to‐event analysis (LgRTTS) than with the comparison of incidence proportions (BGTTS) with the while‐at‐risk strategy for intercurrent events because it potentially suffers from immortal time bias.

**TABLE 5:** Agreement of true positives between analyses (illustrative case study). Statistical alerts classified as true or potentially true positives by expert review. The colour scale from yellow to green corresponds to the increasing number of true positives.

	BGTTS D1	LgRTTS D1	SPT‐FDR D1	SPT‐Bon D1	SCTTS D1	All TTS D1	BGTTS D2	LgRTTS D2	SPT‐FDR D2	SPT‐Bon D2	SCTTS D2	All TTS D2	BGTTS D1&2	LgRTTS LFU	SPT‐FDR LFU	SPT‐Bon LFU	All TTS	All SPT‐FDR	All SPT‐Bon
BGTTS D1	39																		
LgRTTS D1	39	40																	
SPT‐FDR D1	38	38	44																
SPT‐Bon D1	36	36	36	36															
SCTTS D1	39	40	44	36	90														
All TTS D1	39	40	44	36	90	90													
BGTTS D2	36	37	38	33	45	45	45												
LgRTTS D2	38	39	40	35	54	54	45	54											
SPT‐FDR D2	38	39	41	36	54	54	44	51	59										
SPT‐Bon D2	38	39	41	36	46	46	42	45	46	46									
SCTTS D2	39	40	44	36	83	83	45	54	54	46	87								
All TTS D2	39	40	44	36	83	83	45	54	54	46	87	87							
BGTTS D1&2	39	40	41	36	51	51	44	50	49	46	51	51	51						
LgRTTS LFU	39	40	41	36	48	48	42	47	47	45	48	48	48	48					
SPT‐FDR LFU	39	40	43	36	55	55	44	52	54	46	55	55	51	48	58				
SPT‐Bon LFU	38	38	41	36	45	45	39	44	45	43	45	45	45	45	45	45			
All TTS	39	40	44	36	90	90	45	54	54	46	87	87	51	48	55	45	94		
All SPT‐FDR	39	40	44	36	59	59	45	53	59	46	59	59	51	48	58	45	59	64	
All SPT‐Bon	38	39	41	36	48	48	42	47	48	46	48	48	48	47	48	45	48	48	48

**TABLE 6:** Agreement of true positives between analyses (illustrative case study). All statistical alerts. The colour scale from yellow to green corresponds to the increasing number of true positives.

	BGTTS D1	LgRTTS D1	SPT‐FDR D1	SPT‐Bon D1	SCTTS D1	All TTS D1	BGTTS D2	LgRTTS D2	SPT‐FDR D2	SPT‐Bon D2	SCTTS D2	All TTS D2	BGTTS D1&2	LgRTTS LFU	SPT‐FDR LFU	SPT‐Bon LFU	All TTS	All SPT‐FDR	All SPT‐Bon
BGTTS D1	42																		
LgRTTS D1	42	44																	
SPT‐FDR D1	41	41	50																
SPT‐Bon D1	39	39	39	39															
SCTTS D1	42	44	50	39	133														
All TTS D1	42	44	50	39	133	133													
BGTTS D2	39	41	42	36	58	58	58												
LgRTTS D2	41	43	44	38	71	71	58	71											
SPT‐FDR D2	41	42	46	39	69	69	54	64	77										
SPT‐Bon D2	41	42	44	39	53	53	49	52	53	53									
SCTTS D2	42	44	50	39	116	116	58	71	69	53	138								
All TTS D2	42	44	50	39	116	116	58	71	69	53	138	138							
BGTTS D1&2	42	44	46	39	67	67	56	65	61	53	67	67	67						
LgRTTS LFU	42	43	46	39	57	57	49	55	55	51	57	57	56	57					
SPT‐FDR LFU	42	43	49	39	68	68	52	63	66	53	68	68	63	57	73				
SPT‐Bon LFU	41	41	45	39	51	51	43	49	50	47	51	51	50	51	51	51			
All TTS	42	44	50	39	133	133	58	71	69	53	138	138	67	57	68	51	155		
All SPT‐FDR	42	43	50	39	76	76	55	67	77	53	76	76	64	57	73	51	76	85	
All SPT‐Bon	41	42	45	39	57	57	49	55	56	53	57	57	56	55	57	51	57	57	57

Abbreviations: BGTTS, between group tree‐temporal scan; D1, Dose 1; D2, Dose 2; LFU, Long term follow‐up; LgRTTS, log‐rank based tree‐temporal scan; MedDRA, Medical Dictionary for Regulatory Activities; PT, Preferred term; RZV, recombinant Zoster vaccine; SCTTS, self‐controlled tree‐temporal scan; SPT‐Bon, asymptotic test for stratified person‐time data with Bonferroni correction; SPT‐FDR, asymptotic test for stratified person‐time data with false discovery rate correction; TTS, tree‐temporal scan.

The SPT‐FDR detected one additional listed PT compared to BGTTS, LgRTTS and SPT‐Bon after dose 1, likely due to the less stringent multiplicity adjustment (Table 3). The same difference was observed between LgRTTS post dose 2 and the BGTTS in the combined 30‐day periods post dose 1 and dose 2. The LgRTTS has generally less power than the SPT‐FDR explaining the listed event only detected by SPT‐FDR, however the temporal scan allows to focus on a period where the group difference is higher, explaining the listed event only detected by LgRTTS. The BGTTS in the combined doses has also higher power due to the inclusion of data from both doses. The temporal scan in the BGTTS combines both doses in each exposure window and may explain why an alert specific to dose 2 was not detected. The principal stratum strategy for intercurrent events of the BGTTS of combined doses may also be part of the explanation.

#### 5.3.3 Analyses within the 420‐day period after vaccination

In the extended follow‐up analyses 420 days post dose 1, the same 15 listed PTs were detected by the LgRTTS and the SPT‐Bon (Tables 2 and 3). One additional listed PT was detected by the SPT‐FDR, potentially due to lower multiplicity adjustment. The PPV of the SPT‐FDR was 68% with five additional true positives compared to LgRTTS, and the PPV of SPT‐Bon was 82% with three statistical alerts less than LgRTTS. No statistical alerts were detected at the SOC level only by LgRTTS within 420 days post dose 1. Eight statistical alerts were detected at the HLT level.

#### 5.3.4 Summary and agreement between the three estimands

The SCTTS analyses for the detection of any transient effect within 20 days after each dose captured all true positives (listed PTs or confirmed by expert review) detected by any of the analyses of the other estimands, within 30 days after each dose or within 420 days after dose 1, including SPT methods (Tables 3 and 4).

**FIGURE 1:**
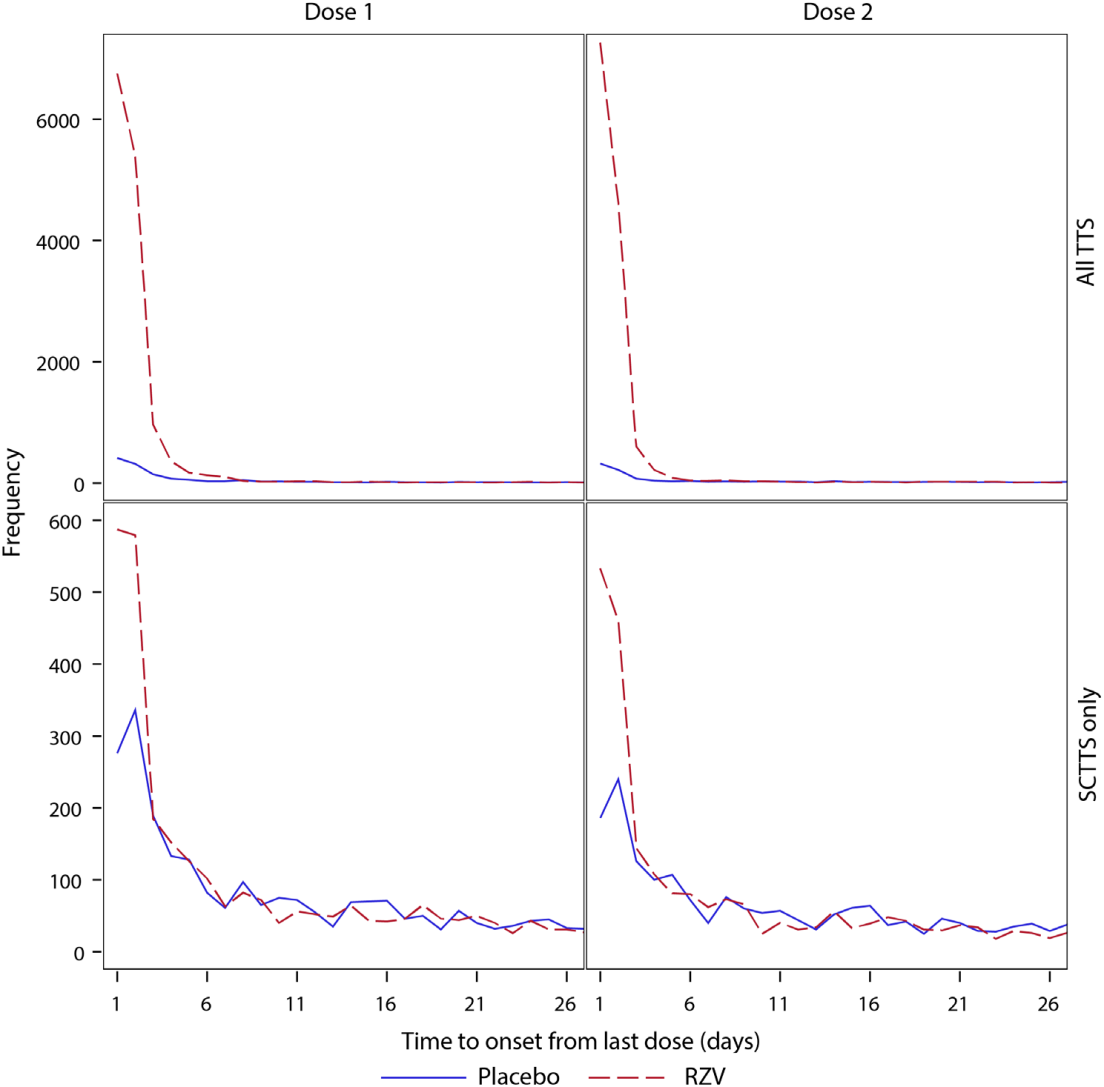
Time‐to‐onset distributions for statistical alerts generated by SCTTS only and by all TTS methods in each group, by dose. SCTTS only, statistical alerts generated only by self‐controlled tree‐temporal scan; All TTS, statistical alerts common to all scan statistical methods. RZV, recombinant zoster vaccine.

The SCTTS method captured all 155 statistical alerts detected by all scan statistical methods in this case study (Table 6). A total of 81 statistical alerts were only detected by the self‐controlled analyses. The higher Se of the SCTTS is illustrated by the distributions of TTOs in the placebo group for statistical alerts only detected by the SCTTS, with high frequency of events with low TTOs (Figure 1).

Overall, nine statistical alerts were only detected by the SPT‐FDR and not by scan statistical methods; none were true positives, and five were potentially true positives.

Only one statistical alert from the LgRTTS in the extended follow‐up was not detected by any of the BGTTS analyses within the combined 30‐day periods post dose‐1 and 2 (Table 6). For this alert, the temporal scan of the LgRTTS identified an exposure period of 55 days after dose 1, increasing the number of cases from 23 within 30 days after dose 1 to 28 with all five additional cases in the exposed group.

## 6 SIMULATION STUDY

We simulated data to examine the relative performance of the three scan statistical methods and SPT tests with multiplicity adjustment. We evaluated the capacity to detect true adverse reactions (ARs) and the capacity to keep the number of false positives at a desired level under a wide range of scenarios.

### 6.1 Simulation strategies

The simulation study was based on the real demography, exposure, and follow‐up time characteristics of the pooled participants from the same two pivotal phase III efficacy studies with RZV. AEs were generated from Monte‐Carlo simulations. Under a homogeneous Poisson process, that is, a constant hazard rate, the probability density function of the time between vaccination and the onset of incident events corresponds to a random variable with an exponential distribution.^37^ Details of the TTO simulations are presented in the supplementary material. We performed two sets of Monte‐Carlo simulations, one for persistent, and the other for transient exposure effects. Additionally, the probability of type I error was evaluated by random permutations of the exposure groups in the real data.

#### 6.1.1 Simulation scenarios

Several datasets were generated under different scenarios for both simulation sets: transient or persistent exposure effects. The simulated AEs in each simulation set were defined by the baseline incidence rates (BI), the relative incidence (RI) of the exposure effect, and the period of the exposure effect. Realistic BI and RI levels were based on their distributions in the real data. The AEs were simulated a second time with a hierarchical structure by randomly distributing the simulated outcomes (HLTs) into several sub‐level outcomes (PTs). This distribution was based on the distribution of MedDRA HLTs with similar incidences into three PT sub‐levels in the real safety data from both studies. Only one dose was considered in the simulation study.

In the simulation set of transient effects, positive control AEs were simulated within 28 days after exposure for each combination of BIs (0.001, 0.01 and 1 event per patient‐year), RIs (1.25, 5 and 10) and exposure periods (Days [0–6] and [7–13]), resulting in potentially 90 positive controls (PC), 36 at HLT level and half of them randomly distributed in 54 PCs at PT level.

In the simulation set of persistent effects, the PCs were generated within 420 days after exposure. Combinations were based on BIs (0.0001, 0.01 and 0.1 event per patient‐year), RIs (1.25, 2 and 5) and immediate effect or delayed effect starting at day 60 after vaccination, resulting also in 36 PCs at HLT level and 132 PCs at PT level due to random distribution into PTs for half of the HLTs.

In both simulation sets, the same 36 scenarios were repeated for the negative controls (NC), with RI = 1, generating balanced sets of positive and negative controls. Due to the computational burden, each dataset was simulated 100 times.

#### 6.1.2 Evaluations

The LgRTSS, SPT‐FDR and SPT‐Bon tests were evaluated in both simulations sets. All AEs at all levels in the hierarchical tree (HLTs and PTs) were considered as distinct tests in the multiplicity adjustment of SPT. In addition, the SCTTS and the BGTTS were evaluated for the detection of transient effects.

For the simulation set of persistent exposure effect, the analysis period was 420 days after exposure. The temporal scan of the LgRTTS was performed within 420 days by 60 days of increments and 7 days of shifts. For the simulation set of transient exposure effect, the temporal scans of the BGTTS and the LgRTTS were performed within the 28 days after exposure by 7 days of increments and shifts. For the SCTTS the temporal scan was performed within 21 days after exposure, the last 7 days being the fixed part of the control period.

The performance metrics of each quantitative signal detection (QSD) analysis were estimated in each of the 100 simulated datasets, each of them including the real clinical trial AE data and the simulated AEs. The real AE data were kept in the QSD analyses to maintain a realistic background noise of many correlated outcomes that can influence performance when detecting simulated AEs due to increased multiplicity of testing.

The results of the real AE data were ignored in the estimation of the performance metrics. Se being the proportion of true vaccine‐AE associations detected among all true associations, only simulated AEs with RI >1 were considered in its computation. The specificity (Sp) was estimated as the proportion of simulated NC (RI = 1) detected and PPV was estimated from the proportion of true positives among all simulated AEs detected. The differences in performance between the QSD methods were evaluated by averaging the performance characteristics over the 100 simulation sets. The averaged Se, Sp and PPV for varying significance levels (alpha) between 0.01 and 0.95 were summarised by receiver‐operator characteristics (ROC) and precision‐recall (P‐R) curves.^42^ The probability of type I error was estimated for varying alpha levels as the proportion of permuted datasets with at least one false positive among 250 permutations for the transient effects, and 500 permutations for the persistent effects. The exposure groups were randomly permuted in each dataset to remove group differences.

### 6.2 Simulations results

**FIGURE 2:**
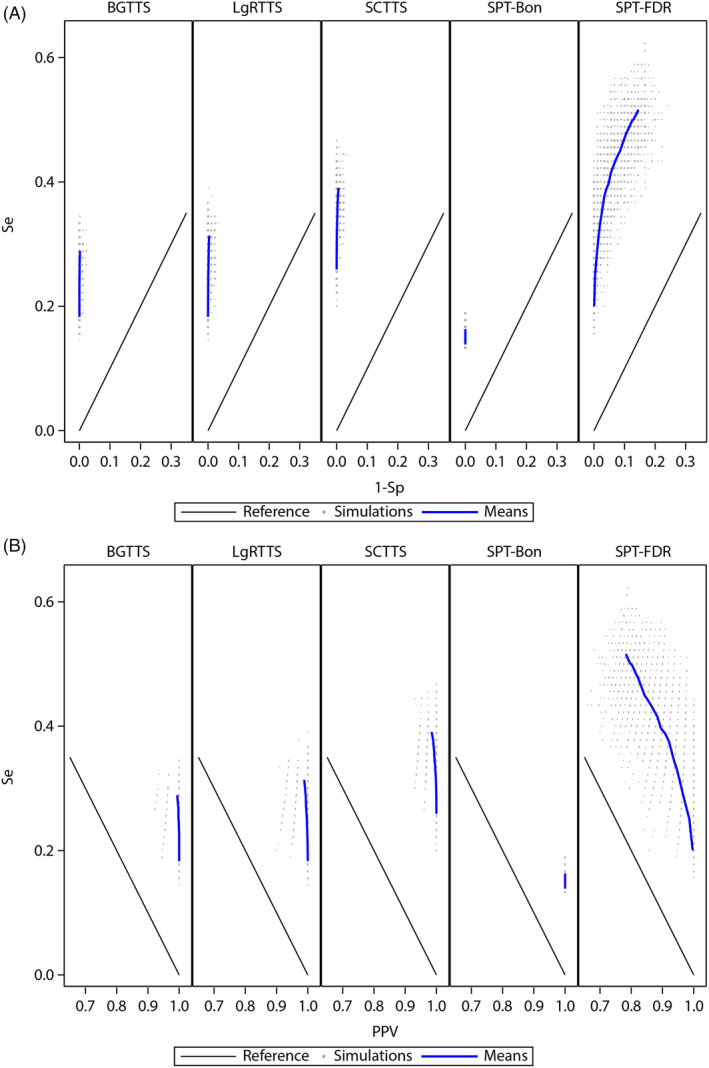
Receiver‐operator characteristics (A) and precision‐recall (B) curves across simulations of transient exposure effect for varying alpha levels (0.01 to 0.95), by quantitative signal detection methods. BGTTS, between group tree‐temporal scan; LgRTTS, log‐rank based tree‐temporal scan; PPV, positive predictive value; SCTTS, self‐controlled tree‐temporal scan; Se, sensitivity; Sp, specificity; SPT‐Bon, asymptotic test for stratified person‐time data^24^ with Bonferroni correction; SPT‐FDR, asymptotic test for stratified person‐time data^24^ with false discovery rate correction.

In the simulation set of transient exposure effects, the Se for each alpha levels was estimated from the 90 simulated PCs and the Sp from the 90 simulated NCs. The ROC and P‐R curves of each method based on mean Se, Sp and PPV across 100 simulations are presented in Figure 2.

The performance metric ranges were small for alpha ranging from 0.01 to 0.95, especially for the PPV and the Sp. The SPT‐Bon showed the lowest Se, 15% for alpha of 0.1, and Sp and PPV equal to 1 for the entire range of alpha, including 0.95. The ROC and P‐R curves of the SPT‐FDR were the closest to the reference lines, indicating a less favourable balance than the other methods between the Se and Sp, and Se and PPV. The variability across simulations was also the highest (see simulation points in Figure 2).

Between the scan statistical methods, the SCTTS showed the most favourable balance between performance metrics, and highest Se, 30% for alpha 0.1. The LgRTTS had higher Se than BGTTS, 22% and 21% respectively for alpha of 0.1, with limited difference in Sp and PPV. Of note, the differences in Se between methods are in line with the results from the case study (Table 2), especially post dose 2 with 29% of Se for SCTTS, 19% for BGTTS and 22% for LgRTTS. In contrast with the case study, the PPV and Sp of the SCTTS remained close to 100%.

**FIGURE 3:**
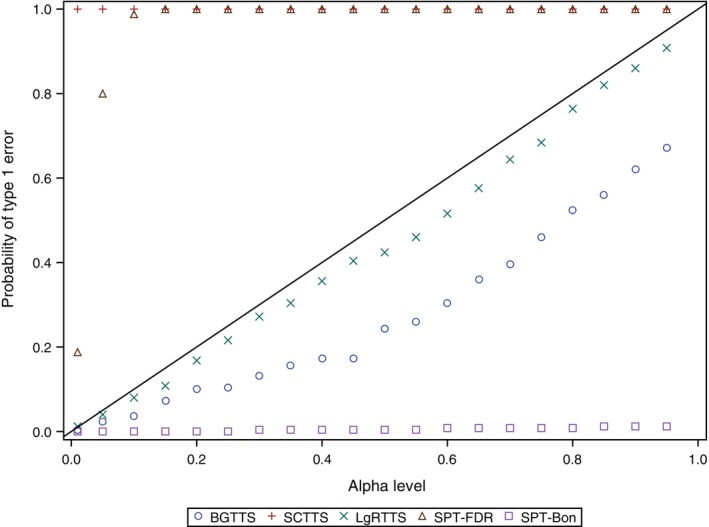
Probability of type I error for varying alpha levels (0.01 to 0.95), by quantitative signal detection methods for transient exposure effect. BGTTS, between group tree‐temporal scan; LgRTTS, log‐rank based tree‐temporal scan; SCTTS, self‐controlled tree‐temporal scan; SPT‐Bon, asymptotic test for stratified person‐time data^24^ with Bonferroni correction; SPT‐FDR, asymptotic test for stratified person‐time data^24^ with false discovery rate correction.

The probability of type I error with SPT‐Bon was much lower than the alpha (Figure 3). The SPT‐FDR reached a probability close to 1 from an alpha of 0.15 and above. As expected, the SCTTS method had a probability of false positives of 1 for all alpha levels, including 0.01, because the permutations between the groups only diluted the temporal clusters due to exposure in the data. The BGTTS method had probabilities consistently lower than the alpha levels. The LgRTTS had probabilities slightly lower than the alpha level but closer than the BGTTS.

**FIGURE 4:**
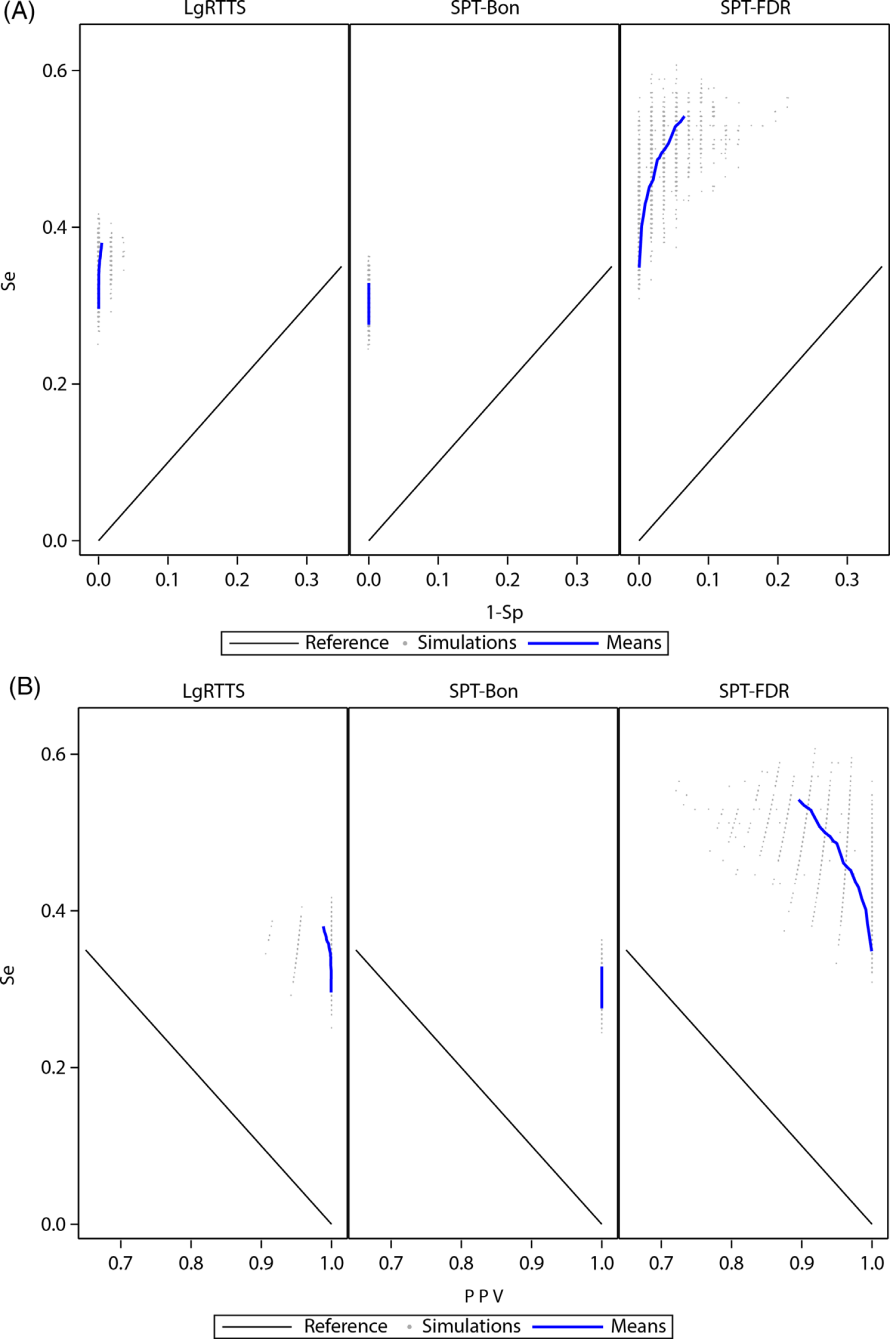
Receiver‐operator characteristics (A) and precision‐recall (B) curves across simulations of persistent exposure effect for varying alpha levels (0.01 to 0.95), by quantitative signal detection methods. LgRTTS, log‐rank based tree‐temporal scan; PPV, positive predictive value; Se, sensitivity; Sp, Specificity; SPT‐Bon, asymptotic test for stratified person‐time data^24^ with Bonferroni correction; SPT‐FDR, asymptotic test for stratified person‐time data^24^ with false discovery rate correction.

**FIGURE 5:**
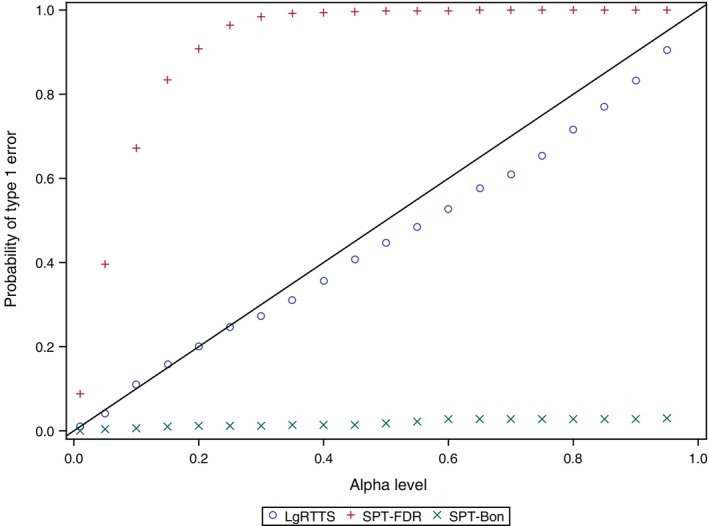
Probability of type I error for varying alpha levels (0.01 to 0.95), by quantitative signal detection methods for persistent exposure effect. LgRTTS, log‐rank based tree‐temporal scan; SPT‐Bon, asymptotic test for stratified person‐time data^24^ with Bonferroni correction; SPT‐FDR, asymptotic test for stratified person‐time data^24^ with false discovery rate correction.

In the simulation set of persistent effect, the Se was estimated from the 168 simulated PCs and the Sp from the 168 simulated NCs. The ROC and P‐R graphs of each method based on mean Se, Sp and PPV across 100 simulations are presented in Figure 4. The performance metric ranges were small for alpha ranging from 0.01 to 0.95, especially for the PPV and the Sp. The SPT‐Bon showed the lowest mean Se, 30% for alpha of 0.1, and Sp and PPV equal to 1 for the entire range of alpha. The SPT‐FDR showed the highest mean Se, 40% for alpha of 0.1, with mean Sp of 0.996 and mean PPV of 0.991. The variability of the Sp and PPV was much higher than of LgRTTS and SPT‐Bon (Figure 4). The mean Se of the LgRTTS for alpha of 0.1 was 32%, with both mean Sp and mean PPV very close to 1. The probability of type I error with SPT‐Bon was much lower than the alpha (Figure 5). The SPT‐FDR was much higher than alpha and reached a probability above 0.99 from an alpha of 0.35 and above. The LgRTTS had probabilities slightly lower than the alpha level.

## 7 DISCUSSION

The quantitative analysis of safety data in clinical trials is a data mining issue aiming at generating statistical alerts for hypothetical safety issues. Statistical alerts generation is only a first step in the detection of safety signals, and statistical alerts are only considered signals after subsequent triaging and validation steps. Safety estimands should integrate the flexibility required for hypothesis generation in the variable/endpoint definitions, in the strategies for intercurrent events and, because the estimand attributes are interrelated, in the statistical methods to generate population‐level summaries. We evaluated the performance of three complementary scan statistical methods in detecting statistical alerts in safety data captured during the clinical development of vaccines. The type II error, that is, failing to detect statistical alerts that result in true safety signals, is therefore the main concern when statistical tests are used for the evaluation of clinical safety data. However, a trade‐off must be found between the risk of type II errors and risk of detecting too many false positives. In this context, adjustment for multiplicity to manage the type I error probability is useful.

Scan statistics have been used for safety signal detection in post‐marketing studies where large numbers of outcomes are tested.^27^, ^41^, ^46^, ^47^ These methods minimise the number of false positives by adjustment for multiple testing using Monte‐Carlo simulations or permutations, without the need for assumptions on the correlation structure between the outcomes. To our knowledge, this is the first study evaluating these three scan statistical methods for safety signal detection in clinical trials, and this is the first time the tree scan is extended to the log‐rank test.

The three methods described herein are theoretically complementary. The SCTTS method is a self‐controlled approach efficient for detecting temporary increases in the risk of an AE after exposure. The BGTTS method can detect differences between two groups in terms of both transient and persistent changes in risk. As this method compares two groups, it can benefit from a RCT design. If the vaccination schedule of the two groups is the same, the different periods of surveillance of AEs (solicited AEs, unsolicited AEs, SAEs or pIMDs) are addressed due to exchangeability between groups. This makes the adjustment for the study effects very important when pooling studies with different designs.

In the SCTTS and BGTTS methods, study participants are considered to be followed for the entire pre‐defined analysis period. Depending on the strategy to address intercurrent events, this may bias the results in each direction. When withdrawn study participant are ignored, events associated with premature participant withdrawal from the study might not be detected as a statistical alert because those cases would not be included in the analysis. When all participants are included, ignoring withdrawals during the analysis, time‐not‐at‐risk is included in the analysis, usually in the control period of the SCTTS, underestimating the baseline risk. By contrast, the LgRTTS method only considers time‐at‐risk and works better for long‐term exposure effects because it addresses premature censoring and allows for longer individual follow‐up.

The tree‐temporal scan methods were evaluated in the pooled data from two pivotal phase III efficacy trials with RZV, in which ~30,000 adults participated, of whom approximately half were exposed to RZV.^38^, ^39^ With this sample size, a self‐controlled case series^48^ (approximation for SCTTS) in the exposed group has 80% power to detect AEs with a 2‐weeks transient RI of 3 within the 28‐days analysis period for background incidence rate (IR) greater than 7/1000 person‐years. A comparison of incidence rates (approximation to SPT) could detect AEs with the same IR. A comparison of proportions (approximation for BGTTS) could detect the same exposure effect for AEs with background IR greater than^38^, ^39^ 8/1000 person‐years. For the log‐rank test, the minimal background IR is 4/1000 person‐years. In the short‐term analyses (30 days), the performances of the BGTTS and SPT were expected to be similar, and the LgRTTS to be higher than the SCTTS method. However, the SCTTS method had the highest Se. The PPV of the SCTTS method was lower, but the price to pay seemed justified given the much larger number of true positives detected and a reasonable total number of statistical alerts. The LgRTTS showed indeed slightly better performance than the BGTTS. Within 420 days after exposure the SPT can detect a RI of 3 for events with background IR greater than 0.24/1000 person‐years, and the log‐rank test an IR greater than 0.27/1000 person‐years. Accordingly, the overall performance of the LgRTTS and SPT methods in the long‐term analysis of the case study was expected to be similar and to mainly depend on the multiplicity adjustment method. The Se of the SPT‐FDR analyses were slightly higher and their PPV slightly lower than their corresponding scan statistical methods likely due to the less strict multiple testing adjustment and their asymptotic nature.

In the case study, unsolicited AEs were collected within 30 days after each dose and only SAEs and pIMDs were actively monitored during the extended follow‐up (until 1 year post dose 2). The apparently limited added value of the LgRTTS method in the long‐term analysis may be attributed to the low number of events reported later than 30 days after each dose. However, the poor performance of the LgRTTS should be tempered by the potentially higher importance of statistical alerts detected in long‐term data, typically SAEs and pIMDs. In addition, long‐term effects are potentially more difficult to detect through post‐marketing pharmacovigilance.

The SCTTS for detecting any transient effects within 20 days after each dose addressed a narrower question of interest than the BGTTS, LgRTTS, and SPT analyses for detecting any transient or persistent effects within 30 days after each dose, yet the SCTTS detected all the true positives detected by the BGTTS, the LgRTTS and the SPT, and many more. When the objective is to estimate what would happen in the same individuals, had the vaccine not been administered, a self‐controlled method may be more relevant than a comparison with a placebo group that still involves injections, especially for the reactogenicity, with a positively skewed TTO distribution. This suggests also that including the SCTTS analysis in the detection of any transient or persistent effects within 30 days after each dose was important despite only covering part of the question of interest.

A few statistical alerts detected by the SCTTS method corresponded to ARs detected for RZV in post‐marketing data. The SCTTS is a case‐only method such as the self‐controlled case series, and these methods are popular in post‐marketing pharmacoepidemiologic studies of vaccines.^49^, ^50^, ^51^, ^52^, ^53^, ^54^, ^55^, ^56^

The review of statistical alerts by safety experts allowed evaluation, to some extent, of the added value of the tree scan with respect to alerts at higher levels than MedDRA PTs (HLT, SOC, pIMDs). Few MedDRA higher level statistical alerts had no corresponding statistical alerts at lower levels in the tree. Of those, only a small proportion were considered true positives by experts, indicating that the added value of this analysis was limited. All tree scan analyses used the MedDRA hierarchy, which may not be ideal for events that may have a similar mechanism but may be manifested in different sections of a terminology (i.e., immune‐related AEs).^57^ Other approaches consider some correlation between outcomes according to a hierarchically structured classification. The Berry‐Berry hierarchical model and its derivatives makes the assumption that AEs are interchangeable within higher levels in the hierarchy.^9^, ^31^, ^32^ This makes these methods also very dependent on the capacity of the hierarchical structure of outcomes to adequately representing semantic distance between concepts from a clinical safety perspective. Other combinations of PTs such as SMQs and internally defined combinations may be more beneficial.

The simulation study allowed a better evaluation of the performance of the methods according to the outcome characteristics. More specifically, the transient and persistent exposure effects were simulated in separate sets. In both sets of simulations, there was little variability in Sp and PPV due to the low number of simulated events and the low power of the tests due to multiplicity adjustments. The very high PPVs in comparison with the case study is potentially explained by the absence of confounding factors and systematic biases in the simulation settings. For this reason, the risk of noise was better evaluated by the probability of type I error estimated from permutations of the exposure groups.

In each simulation set, the FDR‐adjusted SPT test had better Se than the scan statistics methods because of less strict multiplicity adjustment, confirmed by the high probability of type I error. There was an exception for SCTTS at low alpha levels where the Se was the highest, in line with the results from the case study with alpha level of 0.1. The LgRTTS method had the best performance for the detection of persistent effects, with high Se and a probability of type I error corresponding to the pre‐defined significance level. The Se of the BGTTS method was lower than the LgRTTS, as anticipated by the inclusion of time‐not‐at‐risk in the analysis. This is consistent with the difference of Se between those two methods in the post dose 2 analyses of the case study. No difference was observed post dose 1 likely because analyses were less affected by premature censoring. The SCTTS method had good Se to detect transient effects. The group permutations only partly diluted the temporal clusters and therefore did not allow us to estimate its risk of type I error. However, the trade‐off between Se and PPV seemed better than all other methods. The adjustment for multiplicity was well achieved by the LgRTTS and BGTTS methods, even though it proved to be slightly conservative, especially for the BGTTS.

The strengths of our study include the use of a large clinical dataset available from two identically designed clinical trials. On the other hand, it was restricted to one vaccine (RZV) limiting the generalisability of the conclusions. Application of these methods to other studies using the same vaccine would have increased the sample size and may have improved their evaluation. Other potential limitations of this work include the fact that the evaluation was based on general performance indicators without differentiation of the characteristics of the event, for example, local or systemic reactions, short versus long duration, onset, severity, and so forth. Evaluating real data generated across an entire CDP could add useful information and could provide a more comprehensive evaluation of safety signal detection during a clinical development. The evaluation of the LgRTTS suggests it was sub‐optimal in the long‐term analyses in this case study. The method was developed for the detection of long‐term effects and in future work should be evaluated in studies of long‐term treatments and diseases. However, the LgRTTS showed good performance in the short‐term analyses when compared to the BGTTS. An argument could therefore be made based on our analyses that the complementary role and added value of the BGTTS is questionable, and perhaps the other two scan statistical methods may be sufficient in at least some circumstances. Finally, the SCTTS relies on a uniform baseline distribution of the TTO after exposure. This condition may not be completely met during vaccine clinical trials. For example, the probability of reporting during the first week(s) after vaccination is increased due to enhanced monitoring of solicited symptoms. This may be addressed by conditional versions of the method, in which the baseline TTO distribution is estimated from other events or other groups.

Temporality is an important aspect of causality assessment.^58^ The capacity to detect biologically meaningful exposure periods of these tree‐temporal scan statistics methods should be further evaluated. Considering that the SCTTS method does not require a comparison group, it could be tested at the level of an entire CDP by pooling the exposed study participants from all relevant clinical trials (as long as the exposed groups are similar). This method may also be useful for safety monitoring during the course of a study, when exchangeability between groups is not yet achieved. In some projects, additional dimensions of scanning could be investigated, such as age strata for influenza vaccines, to identify sub‐groups with specific AEs.

## 8 CONCLUSIONS

Scan statistics are useful for safety signal detection in clinical trials data (study level or pooled studies). The approach with three complementary methods captured most of the AEs listed in the Reference Safety Information of RZV. All three methods formally adjust for multiple testing of multiple overlapping endpoints without being excessively conservative. The extension of the scan statistical methods to the log‐rank test for time‐to‐event analyses showed some promise and should be further evaluated.

The SCTTS method is of particular interest for signal detection or for signal evaluations of transient exposure effects based on clinical trial data. It may be considered a generalisation of the self‐controlled case series for many safety endpoints, without the need for pre‐specifying the period of potential higher risk. The SCTTS method is also the most convenient for pooled data analyses, as it does not require maintenance of randomisation and consistency in the control groups.

## TRADEMARK STATEMENT

*Shingrix* is a trademark of the GSK group of companies.

## FUNDING INFORMATION

This work was sponsored by GlaxoSmithKline Biologicals SA in all stages of the study conduct and analysis. GlaxoSmithKline Biologicals SA also took responsibility for all costs associated with the development and publishing of the present manuscript.

## CONFLICT OF INTEREST STATEMENT

All authors are employees of the GSK group of companies. All authors hold shares in the GSK group of companies as part of their employee remuneration.

## Supporting information


**Data S1:** Supporting information.

## Data Availability

No new real data were generated in this publication. The clinical trials data mentioned in this publication can be found in the references of the primary publications.^38^, ^39^
