# Supplementary material for: Tree‐temporal scan statistics for safety signal detection in vaccine clinical trials
Source: Pharm Stat. 2024 Apr 15;23(6):813–36. doi: 10.1002/pst.2391 (PMC11602958; doi:10.1002/pst.2391)
Supplement: Supplementary file 1 — Data S1: Supporting information. [file PST-23-813-s001.docx]

# Supplements

### ***Simulating Time-to-Onset***

Let *T_ij_* be the Time-to-Onset (TTO) of adverse eventAE_j_ for subject *i*. Assuming a baseline incidence rate *λ_j_* for AE_j_, a relative incidence *r_j_* for the permanent exposure effect on the risk of AE_j_, and *x_i_* an indicator of exposure for subject *i*, $x_{i}\in\left\{ 0,1 \right\}$. We generated for each subject *i* and for each AE_j_ the *T_ij_* from independent realisations of an exponential distribution: $T_{ij}\sim E(\lambda_{j}r_{j}x_{i})$.

Assuming that the exposure effect has a lag of *u* days, we generated realisations from two independent exponential distributions, $T_{ij}^{1}\sim E(\lambda_{j})$ and $T_{ij}^{2}\sim E(\lambda_{j}r_{j}x_{i})$, such that, by the lack of memory property of the exponential distribution, $T_{ij}=T_{ij}^{1}$ if $T_{ij}^{1}<u$ and $T_{ij}={(T}_{ij}^{2}+u-1)$ otherwise.

Assuming a transient exposure effect that extends between days *a* and *b* following exposure, we generated realisations from three independent exponential distributions, $T_{ij}^{1}\sim E(\lambda_{j})$, $T_{ij}^{2}\sim E\left( \lambda_{j}r_{j}x_{i} \right)$, and $T_{ij}^{3}\sim E(\lambda_{j})$ such that $T_{ij}=T_{ij}^{1}$ if $T_{ij}^{1}<a$, $T_{ij}={(T}_{ij}^{2}+a)$ if $T_{ij}^{1}\geq a$ and ${(T}_{ij}^{2}+a)\leq b$, and $T_{ij}={(T}_{ij}^{3}+b+1)$ if $T_{ij}^{1}\geq a$ and ${(T}_{ij}^{2}+a)>b$.

### ***Stepwise Description of Scan Statistical Methods***

*Self-Controlled Tree-Temporal Scan*

1. Selection of adverse events (AE) with onset date within the analysis period following the exposure. Example: within day0-29 after first dose in the illustrative case study.
2. Classification of each AE within nodes at each level of the hierarchical tree used for the tree-scan. Example: each AE is classified in MedDRA PT nodes, in HLT nodes and in SOC nodes.
3. For each tree node, selection of the first event by individual to create the analysis dataset
4. Simulations to generate many datasets under H0 (no exposure effect). The analysis dataset is reproduced many times with the onset days of each node within each individual simulated from a uniform distribution over the analysis period. Example: 9999 simulated datasets with onset days uniformly distributed within 30 days after exposure.
5. Temporal scan within the original analysis dataset and within each of the simulated datasets.
   1. In each step of the temporal scan, the analysis period is split into an exposure period and control periods before and after the exposure window. Example: an analysis period of 30 days split into an exposure period day5-10 and control periods day0-4 and day11-29.
   2. For each node in the tree, computation of the log likelihood ratio (LLR) test statistic for each step in the temporal scan:

$$LLR=\ln\frac{\left( \frac{a}{a+b} \right)^{a}\left( \frac{b}{a+b} \right)^{b}}{\left( \frac{r}{r+c} \right)^{a}\left( \frac{c}{r+c} \right)^{b}} I\left( \left( \frac{a}{a+b} \right)>\left( \frac{r}{r+c} \right) \right)$$

Where

*a* is the number of node cases with onset in the exposure period,
 *b* is the number of node cases with onset in the control period(s),
 *r* is the length of the exposed period, and
 *c* is the length of the control period(s).
The I( ) is the indication function ensuring that we are looking for an excess risk in the exposure period.

- 1. For each node in the tree, selection of the maximum of LLR over all steps in the temporal scan.

1. Empirical distribution of the Maximum of LLR over all nodes: for each of the original analysis dataset and the simulated datasets, selection of the maximum over all nodes of their maximum of LLR in the temporal scan. The empirical distribution consists of one LLR by simulation, which is the maximum of LLR over all nodes and all steps of the temporal scan.
2. The p-value of each node is determined by positioning its maximum of LLR over the temporal scan from the original data in the empirical distribution created in 6.

*Between Group Tree-Temporal Scan*

1. Selection of adverse events (AE) with onset date within the analysis period following the exposure. Example: within day0-29 after first dose of RZV or placebo in the illustrative case study.
2. Classification of each AE within nodes at each level of the hierarchical tree used for the tree-scan. Example: each AE is classified in MedDRA PT nodes, in HLT nodes and in SOC nodes.
3. For each tree node, selection of the first event by individual to create the analysis dataset
4. Simulations to generate many datasets under H0 (no exposure effect). The analysis dataset is reproduced many times with the exposure group (example: RZV or placebo) of each individual simulated from a Bernouilli distribution with the proportion of exposed individuals (example: RZV) as parameter. Example: 9999 simulated datasets exposure group simulated from a Bernouilli according the proportion of individuals exposed to RZV in original data.
5. Temporal scan within the original analysis dataset and within each of the simulated datasets.
   1. In each step of the temporal scan, the analysis period is limited according to the rules of the scan. Example: only data within 10 days after exposure is kept.
   2. For each node in the tree, computation of the log likelihood ratio (LLR) test statistic for each step in the temporal scan:

$$LLR=\ln\frac{\left( \frac{a}{a+b} \right)^{a}\left( \frac{b}{a+b} \right)^{b}}{\left( \frac{E}{E+C} \right)^{a}\left( \frac{C}{E+C} \right)^{b}} I\left( \left( \frac{a}{a+b} \right)>\left( \frac{E}{E+C} \right) \right)$$

where *a* is the number of node cases in the exposed group,
 *b* is the number of node cases in the control group,
 *E* is the number of exposed subjects, and
 *C* is the number of subjects in the control group.

- 1. For each node in the tree, selection of the maximum of LLR over all steps in the temporal scan.

1. Empirical distribution of the Maximum of LLR over all nodes: for each of the original analysis dataset and the simulated datasets, selection of the maximum over all nodes of their maximum of LLR in the temporal scan. The empirical distribution consists of one LLR by simulation, which is the maximum of LLR over all nodes and all steps of the temporal scan.
2. The p-value of each node is determined by positioning its maximum of LLR over the temporal scan from original data in the empirical distribution created in 6.

*Log-Rank Based Tree-Temporal Scan*

1. Selection of adverse events (AE) with onset date within the analysis period following the exposure. Example: within day0-419 after first dose of RZV or placebo in the illustrative case study.
2. Classification of each AE within nodes at each level of the hierarchical tree used for the tree-scan. Example: each AE is classified in MedDRA PT nodes, in HLT nodes and in SOC nodes.
3. For each tree node, selection of the first event, computation of time to event.
4. For each tree node, computation of the lifetable.
5. Simulations to generate many lifetables for each node under H0 (no exposure effect). The lifetables of each node are reproduced many times. At each timepoint, the events are distributed randomly in the exposure groups (exposed or control) according to a binomial distribution with the number of events as the number of experiments and the proportion of remaining individuals (not censored and without event before the timepoint) in the exposed group among all remaining individuals just before the timepoint as the probability of success. Example: 9999 simulated lifetables for each node.
6. Log-rank statistics according to the temporal scan within the original lifetables and within each of the simulated lifetables.
   1. In each step of the temporal scan, restriction of the lifetables to the timepoints included in the analysis period Example: only timepoints within day60-150 after exposure are kept.
   2. For each node in the tree, computation of the log-rank statistic {Collett, 2003 #467}
   3. For each node in the tree, selection of the maximum of the log-rank statistic over all steps in the temporal scan.
7. Empirical distribution of the Maximum of LLR over all nodes: for each of the original lifetables and the simulated lifetables, selection of the maximum over all nodes of their maximum of log-rank statistic in the temporal scan. The empirical distribution consists of one log-rank statistic by simulation, which is the maximum of log-rank statistic over all nodes and all steps of the temporal scan.
8. The p-value of each node is determined by positioning its maximum of log-rank statistic over the temporal scan from the original data in the empirical distribution created in step 7.

### ***SAS Code for the Self-Controlled Tree-Temporal Scan***

***************************************************************************************************

Macro name : SCTTS

Version : 5.00

------------------------------------------------------------------------------------------------

Revisions :

Versions Date Author

1.00 21AUG2017 F. Haguinet

Creation of the macro

2.00 03OCT2017 F. Haguinet

Add conditional analyses on covariates (strata), for instance study effect. The tests are

two-sided because the direction of difference can be different between strata.

Add conditions on taking only subjects with sufficient follow-up for the considered

analysis period.

Change parameters to define the scanning of the analysis period: a minimum control period

at the end of the analysis period, a range for the risk windows, and the risk period to be

scanned. The max length of analysis period is the end of the range of risk windows plus the

min control period.

3.00 20FEB2018 F. Haguinet

Add creation of an ouptut dataset with p-values for each AE and its risk window with maxLLR

Add the observed/expected estimate

Add unidirectionality of the tests: I(c/n > w/T) and I(c>u)

4.00 12APR2018 F. Haguinet

Update of the parameter timewin to allow sequential definitions of risk windows

5.00 29AUG2018 F. Haguinet

Add a parameter to restrict the time scan to risk windows beginning within the first XX% of

the scanned period. Indeed there is no point in testing 2-days windows starting day 30

for instance.

------------------------------------------------------------------------------------------------

Description :

Self-controlled tree temporal scan.

Method presented in "Report of Pilot of self-controlled tree-temporal scan analysis for

Gardasil vaccine." Sentinel CBER/Prism methods.

Two analyses are implemented: unconditional and conditional on the total number of events per

day post vaccination (or in other words the distribution of all events between the risk and

control periods. The empirical distribution of the maximum log-likelihood ratios across all

nodes of the AE hierarchical tree and all windows is built with Monte Carlo simulations of

day of event. Simulations are based on a uniform distributions for the unconditional analyses,

and based on the relative distribution of days of onset across all events for the conditional

analyses. Only first events after exposure are considered.

------------------------------------------------------------------------------------------------

INPUT macro parameters :

in_dta : input dataset containing variables [pid &ae_vars. aedate exdate].

wherecl : where clause in the input dataset. For instance to select the relevant studies and

the treated group.

ae_vars : variables of the hierarchical AE tree. The variables should be sorted from the most

granular to the less. (should be character) Example in MedDRA: PT SOC.

an_start : start day of the risk period to be scanned. Default=0, day of exposure.

an_end : end day of the risk period to be scanned.

timewin : range of lengths of risk windows after exposure in days. The parameters timewin,

an_start, an_end winshft and minctrl should be compatible. Example of list: 7-28-7, meaning 7 to 28 by 7.

winshft : shift interval to be considered when moving the risk windows within the risk period.

rest_pct : percentage for the restriction to the first XX% of the windows of the same length

within the scanned period.

minctrl : Minimum control period at the end of each analysis period. Default=14

min_evt : Minimun of occurences for an event to be analysed

cond : conditional version of the test if Y, unconditional otherwise.

covars : when not empty, the tests are further conditionned by values of covars. For instance

the study identifier. This is applicable only for the conditional version of the test.

In the unconditional version constant covariates have no effect because the tests are

self-controlled.

nb_simul : Number of simulations. At least 1000 recommended.

INPUT macro variables :

None

INPUT txt in U:/data :

None

INPUT datasets :

&in_dta. containing variables [pid: subject identifier

&ae_vars.: list of variables for the AE tree

aedate: date of event onset (format=date9.)

exdate: exposure date (format=date9.)

termdate: date of right censoring (format=date9.)]

The dataset should include all eligible subjects, even if some have no occurence of any AE.

In the unconditional version, subjects without AE can be excluded.

INPUT formats : none

INPUT macros : none

------------------------------------------------------------------------------------------------

OUTPUT dataset :

&out_res.: p-values for each AE and its risk window with maxLLR

------------------------------------------------------------------------------------------------

OUTPUT macro variable: none

****************************************************************************************************;

**%macro** sctts (in_dta=,

out_res=p_val_sc,

wherecl=,

ae_vars=,

an_start=**0**,

an_end=**41**,

timewin=**2**-**28**-**2**,

winshft=**2**,

rest_pct=**20**,

minctrl=**14**,

min_evt=**7**,

cond=Y,

covars=,

nb_simul=**10**);

data in_dta;

set &in_dta. (where=((termdate-exdate) GE &an_end. %if %length(&wherecl.) gt **0** %then %do; and (&wherecl.) %end;));

covall_=**1**;

run;

%if &cond.=Y %then %do;

%let covars_=covall_ &covars.;

%let nb_cov=%sysfunc(countw(&covars_.));

%end;

%else %do;

%let covars_=covall_;

%let nb_cov=1;

%end;

%let nb_lvl=%sysfunc(countw(&ae_vars.));

%* Definition of risk windows ;

data rskwinds;

%do p=%scan(&timewin.,**1**,-) %to %scan(&timewin.,**2**,-) %by %scan(&timewin.,**3**,-);

win_id=**0**;

rskend=**0**;

rskstart=**0**;

do while ((rskend+&winshft.) le (&an_end.-&minctrl.));

rskstart=&an_start.+win_id*&winshft.;

rskend=rskstart+&p.-**1**;

win_id+**1**;

if rskstart le round(&an_end.*&rest_pct./**100**) then output;

end;

%end;

run;

proc sql; select count(*) into:nb_win from rskwinds; quit;

%let nb_win=&nb_win.;

proc sql;

select rskstart into:rskstart1 - :rskstart&nb_win. from rskwinds;

select rskend into:rskend1 - :rskend&nb_win. from rskwinds;

quit;

data win_id;

set rskwinds;

length period $30.;

win_id=_n_;

%do w=**1** %to &nb_win.;

if win_id=&w. then period=STRIP("&&rskstart&w.. - &&rskend&w..");

%end;

run;

%* General selection of cases within the analysis period;

proc sql;

create table cases_ as

select distinct *,aedate-exdate as onset_d format=**4.**

from in_dta (where=(not missing(%scan(&ae_vars.,**1**))))

where (EXDATE+&an_start.) le aedate le (EXDATE+&an_end.);

quit;

proc sql;

drop table lr;

create table lr

(simul_id num,tree_lvl num,ae num,win_id num, llr num);

quit;

%* IDs for Covariates strata ;

proc sort data=cases_;

by &covars_.;

run;

data cases(drop=&covars_.);

set cases_ nobs=tobs;

by &covars_.;

attrib cov_id format=**3.** length=**3**;

retain cov_id(**0**) ;

if first.%scan(&covars_.,&nb_cov.) then cov_id+**1**;

if _N_=tobs then call symput('nb_strat',strip(put(cov_id,**3.**)));

run;

%let covars_=cov_id;

%put nb_strat=&nb_strat.;

%if &cond.=Y %then %do;

data onsetlst;

attrib onset_d format=**4.** length=**4**;

do onset_d=&an_start. to &an_end.;

output;

end;

run;

%end;

%* Keep only the first (incident) event per AE for each tree level;

%do l=**1** %to &nb_lvl.;

proc sort data=cases nodupkey out=cases__&l.;

by pid %scan(&ae_vars.,&l.) aedate;

run;

data cases_&l. (drop=aedate rename=(%scan(&ae_vars.,&l.)=ae));

set cases__&l.(keep=&covars_. pid %scan(&ae_vars.,&l.) onset_d aedate where=(not missing(%scan(&ae_vars.,&l.))));

attrib onset_d format=**4.** length=**4** tree_lvl format=**3.** length=**3**;

by pid %scan(&ae_vars.,&l.) aedate;

tree_lvl=&l.;

if first.%scan(&ae_vars.,&l.) then output;

run;

%* Conditional distribution of TTO determined form the most granular level of the tree ;

%if &cond.=Y and &l.=**1** %then %do;

%do s=**1** %to &nb_strat.;

proc sql;

create table totstr&s. as select distinct onset_d,count(*) as tot_nodes from cases_&l. where cov_id=&s.;

select coalesce(fre_day/tot_nodes,**0**) into:p_lst_&s. separated by ','

from onsetlst as a left join totstr&s. as b on a.onset_d=b.onset_d

left join (select distinct onset_d,count(*) as fre_day from cases_&l. where cov_id=&s. group by onset_d) as c on a.onset_d=c.onset_d;

drop table totstr&s.;

quit;

%put list of probabilities in strata &s.: &&p_lst_&s..;

%end;

%end;

%end;

data allnodes_;

set %do l=**1** %to &nb_lvl.; cases_&l. %end;;

run;

proc sql;

create table allnodes__ as

select *,count(*) as tot_ae

from allnodes_

group by cov_id,tree_lvl,ae;

quit;

data allnod_id(drop=tot_ae);

set allnodes__(where=(tot_ae ge &min_evt.));

run;

%* ID for AEs;

proc sort data=allnod_id;

by tree_lvl ae onset_d;

run;

data allnodes(drop=ae) ae_id(keep=ae_id tree_lvl ae);

set allnod_id;

by tree_lvl ae onset_d;

attrib ae_id format=**4.** length=**4**;

retain ae_id(**0**);

if first.ae then do;

ae_id+**1**;

output ae_id;

end;

output allnodes;

run;

%* Simulations for the distribution of the maxLLR under H0: uniform disribution of each case in the analysis period;

proc sort data=allnodes out=sortnodes;

by &covars_. ae_id onset_d;

run;

%do i=**1** %to &nb_simul.;

%if &i.=**1** %then %do;

data simcase;

attrib onset_d format=**4.** length=**4**;

attrib simul_id format=**6.** length=**6**;

set sortnodes;

simul_id=&i.;

run;

%end;

%else %do;

data tmpcase;

attrib onset_d format=**4.** length=**4**;

attrib simul_id format=**6.** length=**6**;

set sortnodes;

simul_id=&i.;

%if &cond.=Y %then %do;

%do s=**1** %to &nb_strat.;

if cov_id=&s. then onset_d=rantbl(**0**,&&p_lst_&s.**.**)-**1**;

%end;

%end;

%else %do;

onset_d=floor(ranuni(**0**)*(&an_end.-&an_start.+**1**));

%end;

run;

proc sort data=tmpcase;

by &covars_. ae_id onset_d;

run;

proc append base=simcase data=tmpcase; run;

%end;

%end;

%* Total number of cases (all AE) and Number of cases in exposure period. Needed for the conditional tests ;

data _C(keep=simul_id &covars_. tree_lvl C T %do w=**1** %to &nb_win.; z_&w. w_&w.%end;)

_u(keep=simul_id &covars_. tree_lvl ae_id n %do w=**1** %to &nb_win.; c_&w. %end;);

set simcase;

by simul_id &covars_. tree_lvl ae_id onset_d;

retain C(**0**) %do w=**1** %to &nb_win.; z_&w.(**0**) c_&w.(**0**) %end; n(**0**);

T=&an_end.-&an_start.+**1**;

%do w=**1** %to &nb_win.; w_&w.=&&rskend&w.**.**-&&rskstart&w.**.**+**1**; %end;

if first.tree_lvl then do;

C=**0**; %do w=**1** %to &nb_win.; z_&w.=**0**; %end;

end;

if first.ae_id then do;

n=**0**; %do w=**1** %to &nb_win.; c_&w.=**0**; %end;

end;

C+**1**;

n+**1**;

%do w=**1** %to &nb_win.; if &&rskstart&w.**.** le onset_d le &&rskend&w.**.** then do; z_&w.+**1**; c_&w.+**1**; end; %end;

if last.ae_id then output _u;

if last.tree_lvl then output _C;

run;

proc sql;

drop table simcase;

create table smy_sim as

select a.*,b.C, b.T %do w=**1** %to &nb_win.;

, b.w_&w.

%if &cond.=Y %then %do;

,b.z_&w., a.n*b.z_&w./b.C as u_&w.

,case when a.c_&w.=**0** then (b.C-a.c_&w.)*LOG((b.C-a.c_&w.)/(b.C-(a.n*b.z_&w./b.C)))

else a.c_&w.*LOG(a.c_&w./(a.n*b.z_&w./b.C)) +

(b.C-a.c_&w.)*LOG((b.C-a.c_&w.)/(b.C-(a.n*b.z_&w./b.C))) end as llr_&w.

%end;

%else %do;

,case when a.n=**0** or (a.c_&w./a.n) le (b.w_&w./b.T) then **0**

when a.c_&w.=**0** then - (a.n-a.c_&w.)*log((b.T-b.w_&w.)/b.T)

when (a.n-a.c_&w.)=**0** then - a.c_&w.*log(b.w_&w./b.T)

else a.c_&w.*log(a.c_&w./a.n)+ (a.n-a.c_&w.)*log((a.n-a.c_&w.)/a.n)

- a.c_&w.*log(b.w_&w./b.T) - (a.n-a.c_&w.)*log((b.T-b.w_&w.)/b.T) end as llr_&w.

,(a.c_&w./a.n)/(b.w_&w./b.T) as O_E_&w.

%end;

%end;

from _u as a left join _C as b

on a.simul_id=b.simul_id and a.tree_lvl=b.tree_lvl and a.&covars_.=b.&covars_.;

quit;

%if &cond.=Y %then %do;

proc sql;

create table smy_sim_cond as

select simul_id, ae_id %do w=**1** %to &nb_win.;, sum(llr_&w.) as llr_&w., sum(c_&w.) as c_&w., sum(u_&w.) as u_&w. %end;

from smy_sim

group by simul_id, ae_id;

quit;

data smy_sim;

set smy_sim_cond ;

%do w=**1** %to &nb_win.;

O_E_&w.=c_&w./u_&w.;

if **0** lt O_E_&w. le **1** then llr_&w.=**0**;

%end;

run;

%end;

data llr_ae dta_llr;

set smy_sim;

length risk_win $**200** O_E $**100**;

llr=max(%do w=**1** %to &nb_win.; llr_&w., %end; **0**);

output llr_ae;

if simul_id=**1** then do;

risk_win='';

O_E='';

%do w=**1** %to &nb_win.;

if llr_&w.= llr then do;

risk_win=strip(risk_win!!" [&&rskstart&w..-&&rskend&w..]");

O_E=strip(O_E!!" "!!put(O_E_&w.,**8.2**));

end;

%end;

output dta_llr;

end;

run;

proc sql;

create table llr_max as

select distinct simul_id, MAX(llr) as llr

from llr_ae

group by simul_id;

quit;

proc sort data=llr_max;

by descending llr;

run;

data llr_max;

set llr_max;

rank=_N_;

run;

proc sql;

select count(*) into:den_p from llr_max;

quit;

%let den_p=&den_p.;

%put den_p=&den_p.;

data lr_sc_rnkd;

set dta_llr llr_max;

run;

proc sort data=lr_sc_rnkd;

by descending llr ae_id;

run;

%* Computing p-values based on the empirical distribution under H0;

data sc_p_val(keep=ae_id risk_win O_E llr p_val minp_val);

set lr_sc_rnkd;

by descending llr ae_id;

retain minp_val(**0**) p_val(**0**) nb_detect(**0**) rank_(**0**);

if missing(ae_id) then rank_=rank;

if not missing(ae_id) then do;

nb_detect+**1**;

p_val=rank_/&den_p.;

if nb_detect=**1** then minp_val=p_val;

output;

end;

run;

proc sql;

create table lr_sc_dec as

select a.*,b.tree_lvl as tree_id,b.ae

from sc_p_val as a left join ae_id as b

on a.ae_id=b.ae_id

order by a.p_val;

quit;

data &out_res.(drop=tree_id);

set lr_sc_dec(drop=ae_id);

length tree_lvl $**15**;

%do l=**1** %to &nb_lvl.;

if tree_id=&l. then tree_lvl=strip("%scan(&ae_vars.,&l.)");

%end;

run;

proc sql;

drop table in_dta;

drop table rskwinds;

drop table win_id;

%do l=**1** %to &nb_lvl.;

drop table cases_&l.;

drop table cases__&l.;

%end;

drop table cases;

drop table lr;

drop table allnodes_;

drop table allnodes__;

drop table onsetlst;

drop table covs;

drop table allnodes;

drop table sortnodes;

drop table tmpcase;

drop table _C;

drop table _u;

drop table smy_sim;

drop table smy_sim_cond;

drop table lr_sc_rnkd;

drop table dta_llr;

drop table llr_ae;

drop table llr_max;

quit;

**%mend** sctts;
